# The new genus *Pheude* (Coleoptera, Curculionidae, Cossoninae) with description of a new species from mainland China

**DOI:** 10.3897/zookeys.466.8032

**Published:** 2014-12-18

**Authors:** Youssef M. Omar, Runzhi Zhang, Steven R. Davis

**Affiliations:** 1 CAS Key Laboratory of Zoological Systematics and Evolution, Institute of Zoology, Chinese Academy of Sciences, Beijing 100101, China; 2 State Key Laboratory of Integrated Management of Pest Insects and Rodents, Beijing 100101, China; 3 Plant Protection Department, Faculty of Agriculture, Assiut University, Assiut, Egypt; 4 Division of Entomology, Natural History Museum, and Department of Ecology & Evolutionary Biology. 1501 Crestline Drive-Suite #140 University of Kansas Lawrence, Kansas 66049-4401, USA

**Keywords:** Dryotribini, new species, *Pheude punctatus*, China, key to genera

## Abstract

A new weevil, *Pheude punctatus* **gen. et sp. n.**, of the tribe Dryotribini in Cossoninae, is described from Guangdong Province, South China. It differs from the related genera *Dryotribus* Horn and *Microtribodes* Morimoto in having antennae with seven articles and a distinct scutellum, and from *Ochronanus* Pascoe and *Stenomimus* Wollaston in having long antennae, a rostrum with a medio-longitudinal furrow beginning at the posterior margin of the eyes and extending approximately midway on the rostrum, and a moderately elevated, medio-longitudinal carina extending the full length of the pronotum. Other diagnostic characters and illustrations are provided. A key to the genera of Dryotribini known from China is given.

## Introduction

Cossoninae are small to medium-sized, primarily wood-associated weevils with a worldwide distribution. They are represented in China by six tribes, 32 genera, and 72 species (YMO unpublished data based on collection at Institute of Zoology, Chinese Academy of Sciences, Beijing). All native genera are widely distributed in China except for the monotypic *Muschanella* Folwaczny, 1964 (Folwaczny 1964) and *Microtribodes* Morimoto, 1973 (Morimoto 1973) which are known only from Zhejiang Province (East China) and Taiwan, respectively. In this study, we describe a new genus and species of Dryotribini LeConte, 1876 from Guangdong Province (South China). Species in this tribe are coarsely sculptured, have an elongate, apically subcylindrical rostrum, funicle with five, six or seven articles, head small with slight post-ocular constriction, dorso-lateral eyes, visible or obscure scutellum, and slender tibiae (LeConte 1876; Voss 1955; Konishi 1962; Decelle and Voss 1972; Folwaczny 1973); the new genus is exceptional in that the elytra narrow slightly from base to apex, whereas they do not narrow apically in the other genera. Dryotribini contain 49 genera in the Palearctic, Neotropical, Afrotropical, Oriental, Neoguinean and Neozelandic Regions (Alonso-Zarazaga and Lyal 1999, regional nomenclature from Cox 2001). In China, Dryotribini are represented by *Dryotribus* Horn, 1873, *Microtribodes*, *Ochronanus* Pascoe, 1885 and *Stenomimus* Wollaston, 1873 (Csiki 1936; Zhang 1992; Alonso-Zarazaga and Lyal 1999; Kojima and Morimoto 2004).

## Materials and methods

The type specimens are deposited in the Institute of Zoology, Chinese Academy of Sciences, Beijing, China. Observations were made with a Zeiss Semi SV 11 stereomicroscope. Habitus photographs were taken by Micropublisher 5.0 RTV digital camera model: MP5.0-RTV-CLR-10A-color 10 BIT, attached to a Zeiss Stereomicroscope Discovery V12. SEM images were captured using a LEO 1550 FESEM.

Measurements were taken using an ocular micrometer and are defined using the following abbreviations: ACL – antennal club length; ACW – antennal club width; AFL – antennal funicle length; AL – antennal length; ASL – antennal scape length; BL – body length; EL – elytral length; EWB – elytral width at base; EWW – elytral width at widest part; PL – pronotal length; PW – pronotal width (widest part); RL – rostral length (excluding mandibles); RWA – rostral width at apex; RWB – rostral width at base.

Measurements were taken as follows: antennal club width measured at the widest part of the club; body length measured in lateral view from the apex of the elytra to the anterior end of the rostrum; elytral length measured in lateral view starting from the base to the apex; pronotal length measured along the median line; rostral length measured in lateral view from the anterior edge of the eyes to the apex. Funicular articles are enumerated beginning with the pedicel and including all articles before the club. On the elytra, intervals and striae are numbered beginning from the suture and extending laterally. Hind wing terminology follows Zherikhin and Gratshev (1995).

The new genus was compared to the following available identified genera in the National Zoological Museum in the Institute of Zoology, Chinese Academy of Sciences, Beijing, China: *Dryotribus*: 8 ♀ (25 VII 1957) Shandong province; 1 ♀ (17 X 1977) Guangdong province; *Stenomimus*: 1 ♂ (20 V 1938), 2 ♀ (5 III 1952), 6 ♀ (7 III 1952) Guangxi Province; *Ochronanus*: 1 ♂ (20 V 1938) Guangxi Province, China.

## Taxonomic treatment

### Pheude

Taxon classificationAnimaliaColeopteraCurculionidae

Omar & Zhang gen. n.

http://zoobank.org/9AD0B374-A96F-4246-B200-4F15EA9111EF

Figs 1–4 , 5–9 , 10–13 , 14–23 , 24–32 , 33–38

#### Type species.

*Pheude punctatus* Omar & Zhang, here designated.

#### Diagnosis.

Rostrum nearly parallel-sided, rostrum without any keel ventrally, longer than wide (more than 2 × width), with longitudinal furrow dorsally; antenna inserted at basal one-third of rostrum; scape extending beyond hind margin of eye, funicle with seven articles; pronotum longer than wide, base bisinuate, with longitudinal median crest from base to apex; scutellum visible; apical margin of elytra expanded and lower than level of venter, elytral apical margin gently rounded and flattened; third tarsomere entire.

#### Description.

*Form* slightly arched, widest approximately at elytral humeri, slightly tapered both apicad and caudad.

*Mouthparts*. Maxilla (Fig. 14) with 3-segmented palpus, basal two segments each with a single lateral seta; stipes and palpiger each with a single lateral, large seta; galeo-lacinial complex with large, paddle-shaped setae along mesal margin; elongate, slender setae along antero-mesal margin of lacinia. Labium (Fig. 15) with 2-segmented palpus; basal segment with one lateral seta; prementum with two lateral setae on both sides; postmentum with two setae before latero-distal margin on ventral side. Mandibles falcate, left mandible (Fig. 16) with one tooth and molar region, right mandible (Fig. 17) with two teeth and molar region.

*Proventriculus* as in Figure 18.

*Rostrum* longer than broad, punctures with minute suberect setae, with large, deep, longitudinal furrow beginning behind eyes and extending to point of antennal insertion, forming slight cleft in rostrum; point of antennal insertion at basal 1/3 of rostrum; scrobe well-defined, deep, dorsal margin directed towards middle of eye but not touching eye, subsequently extending ventrally below eye.

*Antennae* long, stout; scape: clavate, extending slightly beyond hind margin of eyes; funicle with seven articles; article one (pedicel) longer than others, as well as longer than own width; article two small, shorter than others and shorter than own width; club with three articles, appearing to have four with apical constriction, shorter than funicle.

*Head* small, strongly constricted behind eyes; frons as broad as base of rostrum, with long longitudinal furrow extending midway on rostrum. Eyes oval, strongly convex.

*Pronotum* longer than wide, constricted behind apex.

*Scutellum* visible, deeply sunken, subcircular, finely punctured.

*Elytra* wider than pronotum, transversely concave immediately after antero-dorsal margin; basal margin forming transverse keel from sutural interval to humeri. Humeri umbonate, truncate. Intervals elevated from base to apex; striae wider than intervals, with deep circular punctures, distance between punctures ca. 1.5–2.0 × puncture diameter; apex of elytra (from declivity to apex) expanded laterally and extending slightly below level of abdomen; apices gently rounded and slightly upturned.

*Hindwings* (Fig. 23) slender, lacking jugal area (anal lobe); Rr slender, abbreviated, not reaching rcm; rc absent; 1rs triangular and larger than 2rs; R3 present, forming a very thin, sclerotized stripe; Cu_1_ not reaching posterior margin of wing; r-m absent; A simple, other anal veins absent.

*Mesothorax* (Fig. 20). Mesonotum typical of other cossonines; axillary cord enlarged, lateral margins rounded.

*Metathorax* (Fig. 21). Metanotum with metascutum reaching posterior margin of notum; scutellar groove reaching posterior margin of notum; allocrista angular at antero-mesal angle.

*Thoracic sterna* punctured throughout, distance separating punctures ~1.0–2.0 × puncture diameter; mesoventrite relatively small, coxae separated by distance of 0.5 × diameter of coxa, with short, straight intercoxal projection; metaventrite long; coxae separated by distance approximately equal to diameter of metacoxa, coxae with medio-transverse furrow (Fig. 35). Metendosternite (Fig. 22): with long, narrow hemiductus; furcal arm narrow, apex bifid; anterior tendons inserted near base of furcal arms.

*Legs.* Femur strong, longer than tibia, entirely punctured; tibiae parallel-sided; protibia with distal comb of setae along inner margin; tarsus with five articles, articles one and two equal, three entire, feebly longer and wider than one and two combined; five slightly curved, glossy.

**Figures 1–4.**
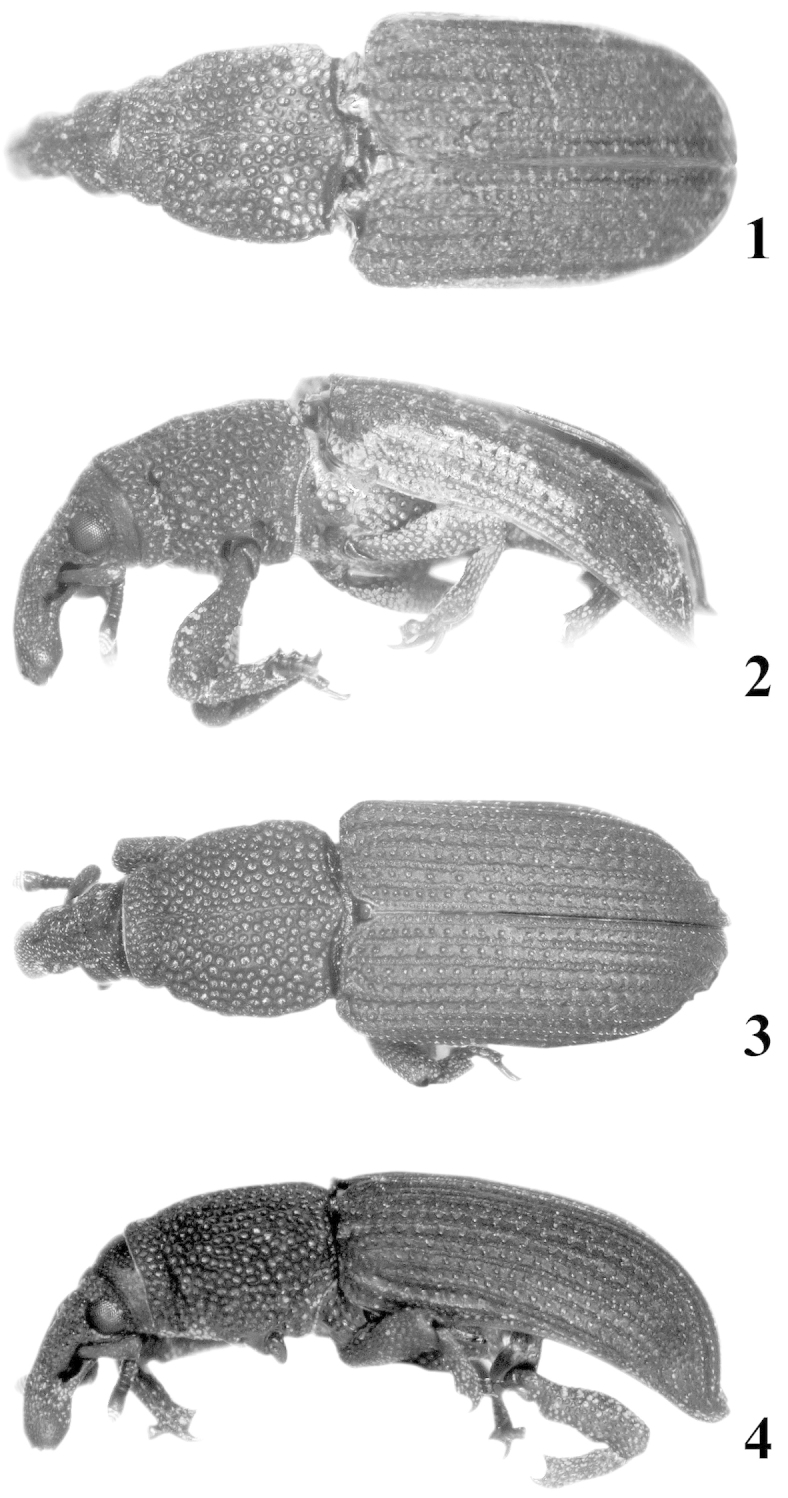
*Pheude punctatus*. **1** Female, dorsal view **2** Female, lateral view **3** Male, dorsal view **4** Male, lateral view.

**Figures 5–9.**
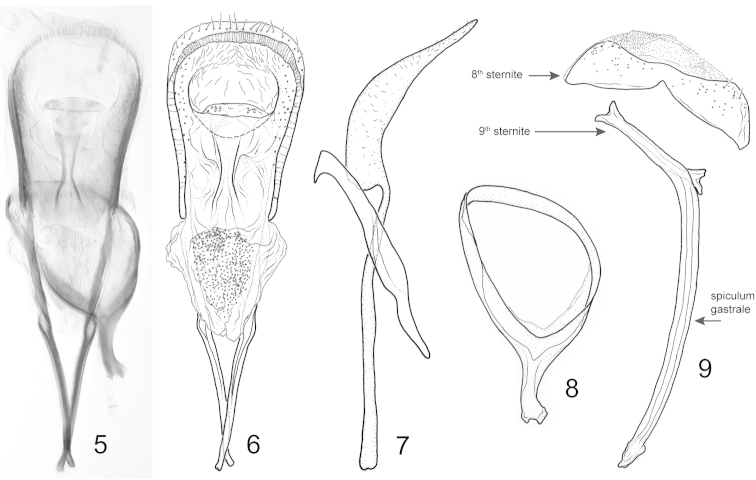
*Pheude punctatus*. Male terminalia. **5–6** aedeagus, dorsal view **7** aedeagus, lateral view. **8** tegmen, ventral view **9** 8^th^ and 9^th^ sternites, dorsal view.

**Figures 10–13.**
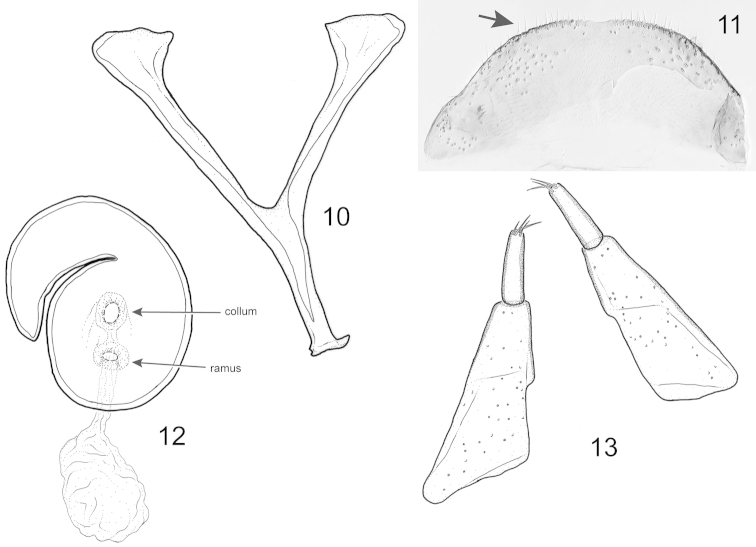
*Pheude punctatus*. Female terminalia and associated tergites. **10** sternite 8 **11** tergite 8 showing short simple setae **12** spermatheca **13** coxites and styli.

**Figures 14–23.**
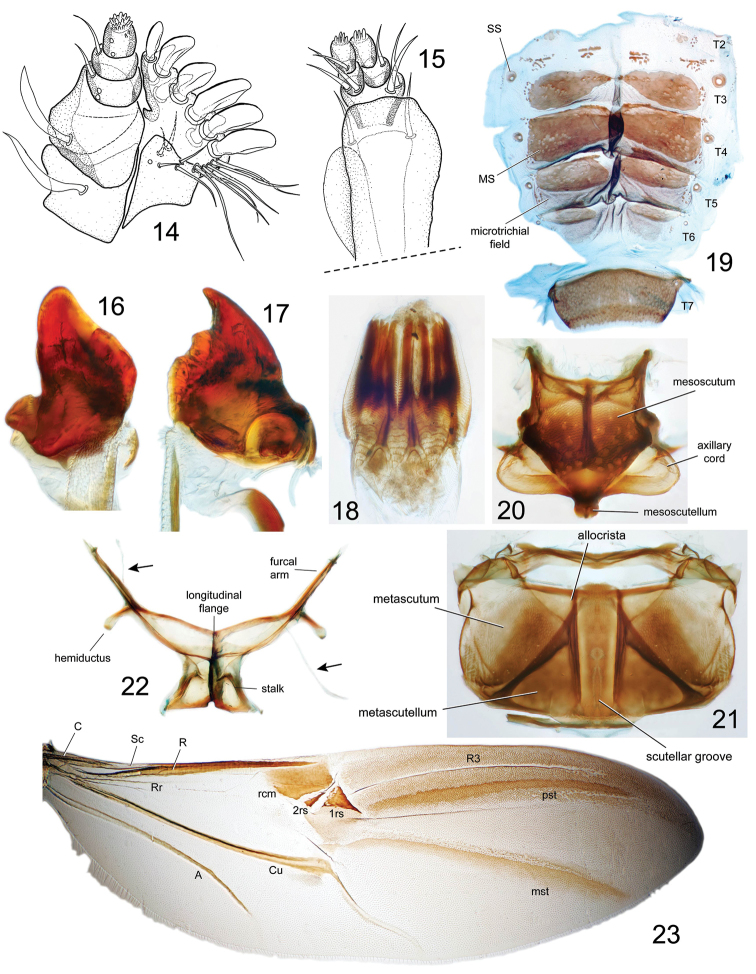
*Pheude punctatus*. **14** maxilla **15** labium **16** left mandible **17** right mandible **18** proventriculus **19** tergum: **MS**=median sclerite **SS**=spiracular sclerite **20** mesonotum **21** metanotum **22** metendosternite showing anterior tendons **23** hind wing: **C**=Costa **Sc**=Subcosta **Rr**=radial recurrent vein **R**=Radius **rcm**=margin of radial cell **2rs, 1rs**=radial sclerites **R3**=3rd radial vein **pst**=postradial stripe **mst**=medial stripe **Cu**=Cubital **A**=Anal.

**Figures 24–32.**
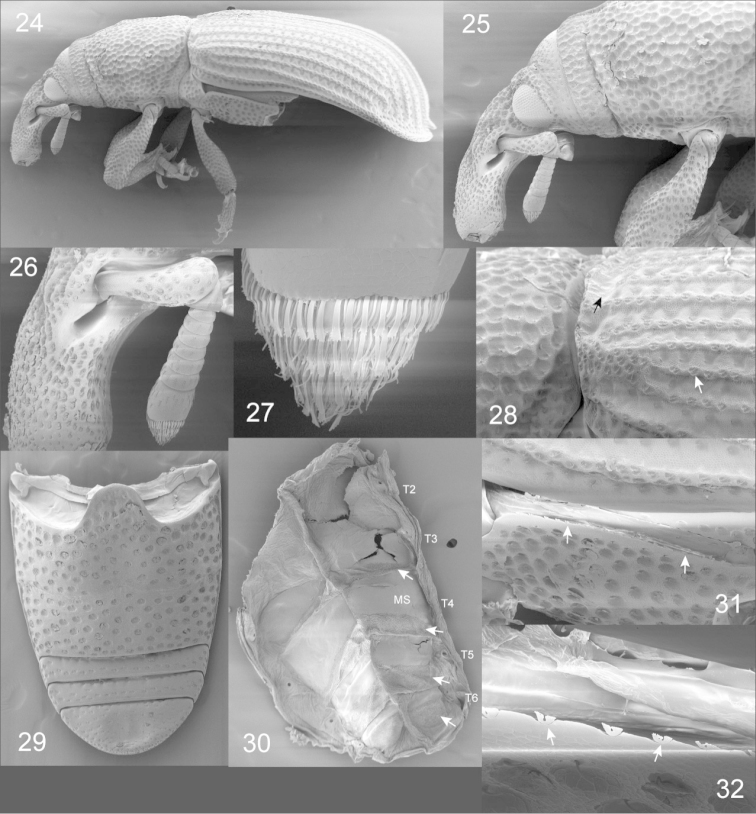
*Pheude punctatus*; SEM micrographs. **24** body, lateral view **25** enlargement of head and anterior portion of prothorax, lateral view **26** enlargement of left antenna and apex of rostrum **27** enlargement of apex of antennal club **28** enlargement of posterior of prothorax and anterior of left elytron, lateral view, showing punctures on elytral intervals and cleft immediately behind antero-dorsal margin of elytron **29** abdominal venter **30** tergum showing microtrichial patches along tergites, posterior to median sclerites: **MS**=median sclerite **31** metathorax, lateral view showing type 2A sclerolepidia (*sensu* Lyal et al. 2006) **32** enlargement of sclerolepidia.

**Figures 33–38.**
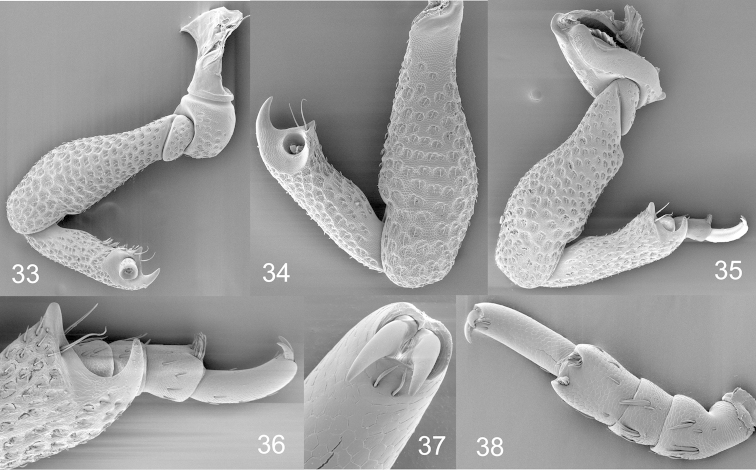
*Pheude punctatus*. **33** fore leg **34** middle leg **35** hind leg **36** distal end of metatibia and metatarsus **37** tarsal claws (pretarsal ungues), ventral view **38** tarsus, ventral view.

#### Etymology.

The name of the new genus honors Pierre Heude (1836–1902), a French Jesuit and zoologist who came to China in 1868. Heude was a cofounder of the first natural history museum in China, and the oldest insect specimens housed in the CAS Institute of Zoology are from this museum, often collected by Octavie Piel. The gender is masculine.

### Pheude punctatus

Taxon classificationAnimaliaColeopteraCurculionidae

Omar & Zhang sp. n.

http://zoobank.org/2C6E297B-A96D-43B3-8DD8-5885CCAA0FFD

Figs 1–4 , 5–9 , 10–13 , 14–23 , 24–32 , 33–38

#### Description.

*Male measurements.* BL: 3.52–4.60 mm; EL: 2.09–2.56 mm; EWB: 1.33–1.65 mm; EWW: 1.38–1.73 mm; PN L: 1.04–1.38 mm; PNW: 1.00–1.25 mm; RL: 0.74–0.89 mm; RWA: 0.34–0.42 mm; RWB: 0.34–0.42 mm; AL: 0.85–1.03 mm; ASL: 0.38–0.44 mm; AFL: 0.34–0.38 mm; ACL: 0.21–0.25 mm; ACW: 0.15–0.19 mm. *Female measurements.* BL: 4.10 mm; EL: 2.00 mm; EWB: 1.52 mm; EWW: 1.55 mm; PN L: 1.14 mm; PNW: 1.12 mm; RL: 0.93 mm; RWA: 0.34 mm; RWB: 0.34 mm; AL: 0.94 mm; ASL: 0.36 mm; AFL: 0.32 mm; ACL: 0.26 mm; ACW: 0.13 mm.

*Integument*. Body densely, deeply punctured throughout (Fig. 24); Color brown to dark brown, opaque, one specimen rusty colored, dull (Figs 1–4).

*Rostrum* long, more than 2 × longer than wide, uneven dorsally, curved from point of anntenal insertion to anterior (apical) fourth of rostrum, apical fourth becoming more linear; dense, elongate, deep punctures throughout, punctures occasionally longitudinally confluent, coarse. Scrobe well-defined, wide, located along basal half of rostrum (Figs 25, 26).

*Antenna* moderately robust, wide; scape with elongate punctures; long, gently widening from base to apex, shorter than funicle and club combined; funicle slightly glossy, chestnut brown, compact, robust, articles three to five approximately equal in size; articles six and seven approximately equal in size, wider than long (Fig. 26); club glossy, chestnut brown (Figs 26, 27); club article 1 longer than others combined and glabrous; club articles 2 and 3 with yellowish, erect setae (Fig. 27), strongly compact, ovate.

*Head* oval, coarse, punctures nearly confluent in various circular and oblong shapes. Eyes dark brown to black, with coarse, convex facets (Fig. 25), widely separated dorsally, located laterally at base of rostrum. Temples swelling.

*Pronotum* with moderately elevated longitudinal carina from base to apex; laterally curved, dorsally convex, with deep, circular punctures, occasionally confluent, unevenly distributed with distance 0.5–1.0 × puncture diameter; each puncture with minute seta off-centered near margin (Fig. 28); basal pronotal margin bisinuate.

*Scutellum* fuscous, glossy, large.

*Elytra* arcuate, basal margin slightly concave, apex gently rounded and emarginate laterally; striae with deep circular punctures, diameter longer than distance between punctures; intervals: with evenly shaped and distributed punctures; punctures with median keel dividing each puncture and with minute setae slightly off-center on keel (Fig. 28); first interval dilated behind declivity to apex, with fine punctures and appressed, minute setae from declivity to apex; intervals four and six connate and fused at declivity (Fig. 24); humeri convex, limited by striae six to eight and intervals six to nine. Sclerolepidia along dorsal margin of metaventrite appearing closer to digitate type 2A (Figs 31, 32), in which sclerolepidia are divided into two distinct lobes, with each lobe divided into several short digits (Lyal et al. 2006).

*Abdominal terga*. Median sclerites developed on T3-6 (Figs 19, 30), small, round spiracular sclerites on all tergites; numerous small sclerites laterally, between median and spiracular sclerites, and scattered medially from T1-2; microtrichial fields present along T3-6 posterior to median sclerites.

*Legs* coarse, femora robust, widening along apical 3/4; tibiae strong, with elliptical, deep punctures; unci large, curved, originating at outer apical angle and small premucro on inner apical angle (Figs 33–36); tarsus article 3 cylindrical, subglabrous; article 5 long, slightly curved (Fig. 38); tarsal claws (pretarsal ungues) simple, joined basally, small (Fig. 37).

*Ventral areas*. Prosternum densely, deeply punctured; distance between procoxae approximately 0.5 × diameter of coxa; procoxal cavities closed, procoxae separated by distance ca. one third of diameter of coxa, positioned close to posterior margin of prosternum; mesoventrite with sparse, deep, circular punctures. Ventrites with sparse, deep, circular punctures, ventrites 1 and 2 slightly elevated, with circular punctures separated by 1–3 × puncture diameter, more convex than other ventrites, posterior margin of ventrite 1 convex medially; 2 slightly narrower than 1; 3 and 4 subequal in width, narrow, sparsely and shallowly punctured; 5 sparsely and shallowly punctured, with large, oval convexity medially.

*Male terminalia and genitalia* (Figs 5–9). Spiculum gastrale broadly curved, with narrow apex; base slender. Eighth sternite not divided, bearing a few setae near postero-lateral margins. Tegmen complete; manubrium short, slightly less than 0.5 × length of tegmen. Penis with median struts slightly longer than median lobe; apical margin of median lobe bearing sparse setae; endophallus (internal sac) bearing numerous minute setae/microtrichia near apex.

*Female terminalia and genitalia* (Figs 10–13). Gonocoxites of typical form; coxites oblong, somewhat quadrate; styli elongate, narrow. Spermatheca with globular base; apex strongly curved. Eighth tergite with slight rounded concavity along margin at middle and row of small setae along apical margin. Eighth sternite with base strongly bifurcate; spiculum short, approximately 0.5 × length of base.

#### Material examined.

**Holotype.** ♂, China: Guangdong Province: Xiancun, Guangzhou; Col. Unknown; VIII 1974; collected from *Aleurites moluccana* (L.) Willdenow; **Paratypes.** 7♂ and 1♀, same data as holotype.

#### Distribution.

Guangdong Province, southern China.

#### Host plant.

The type series was collected from the tree *Aleurites moluccana* (L.) Willdenow (Euphorbiaceae), but it is not known if this is a larval host of the weevil.

#### Etymology.

The specific epithet is a Latin past participle and used to refer to the punctate body of the species.

#### Sexual dimorphism.

No strong differences are apparent between sexes other than the slightly longer and narrower rostrum of the female and the concave first and second ventrites in the male.

## Discussion

*Pheude* is the third monotypic cossonine genus described from China and differs from other oriental cossonine genera, which have been studied by Morimoto (1973), and other Dryotribini genera (e.g. *Lixomimus* Voss; *Cotasteroloeblia* Osella) distributed in adjacent countries (India, Nepal, and Japan) in having the following characters: rostrum nearly parallel-sided, rostrum without any keel ventrally, longer than wide (more than 2 × width), with a longitudinal furrow dorsally; head small; antenna inserted on basal one-third of rostrum; scape extending beyond hind margin of eye, funicle with seven articles; pronotum longer than wide, base bisinuate, with a longitudinal median crest from base to apex; scutellum visible; apical margin of elytra expanded and lower than level of venter, elytral apical margin gently rounded and flattened; third tarsomere entire. Also, *Pheude* differs from the Chinese genera *Muschanella* and *Microtribodes* by the following: *Muschanella* has a wider head, the rostrum widened towards apex, and the antennae inserted slightly before middle of rostrum. *Microtribodes* has an antennal funicle of five articles, the basal half of rostrum with a ventral keel, the antennae inserted before middle of rostrum, and tarsomere 3 bilobed.

The tribe Dryotribini is represented in China by five genera. All these genera are distributed in China and adjacent countries except *Stenomimus*, which is completely Nearctic and Neotropical in distribution, so is presumably introduced into China.

The available distribution of these genera can give an idea that the Chinese cossonine fauna still have so many genera beyond our thinking either to be recorded or to be discovered, so that much more efforts are required for collecting specimens and identification.

### Key to the genera of Dryotribini from China

**Table:** 

1	Funicle with five articles; scutellum minute or indistinct	**2**
–	Funicle with seven articles; scutellum distinct	**3**
2	Rostrum constricted basally, underside without median keel; antenna inserted at middle of rostrum; head strongly constricted behind eyes; scape exceeding hind margin of eye; scutellum indistinct; third tarsomere feebly emarginate	***Dryotribus***
–	Rostrum without constriction, underside with median keel on basal half; antenna inserted before middle of rostrum; head without constriction behind eyes; scape not exceeding hind margin of eye; scutellum small, flat; third tarsomere bilobed	***Microtribodes***
3	Antenna short, scape not reaching eye; rostrum long, curved, without any furrow; pronotum slightly constricted at anterior margin, without median carina	**4**
–	Antenna long, scape extending slightly beyond posterior margin of eye; rostrum with longitudinal median furrow beginning at posterior margin of eyes and extending approximately midway on rostrum, furrow becoming shallower in anterior third; pronotum clearly constricted slightly before anterior margin, with moderately elevated, median longitudinal carina extending from anterior to posterior margin	***Pheude***
4	Eyes oval, not visible in dorsal view; scrobe oblique, running ventrally at base of rostrum; prothorax oblong, with shallow subapical constriction not extending across dorsum; procoxae separated by distance less than one fourth of the diameter of coxa	***Ochronanus***
–	Eyes more rounded, prominent; scrobe with dorsal margin directed to middle of eye; prothorax more triangular, with deep subapical constriction; procoxae separated by approximately half the diameter of coxa	***Stenomimus***

## Supplementary Material

XML Treatment for Pheude

XML Treatment for Pheude punctatus
